# Sensitive and Rapid Detection of the Plasmid-Encoded Colistin-Resistance Gene *mcr-1* in *Enterobacteriaceae* Isolates by Loop-Mediated Isothermal Amplification

**DOI:** 10.3389/fmicb.2017.02356

**Published:** 2017-11-29

**Authors:** Dayang Zou, Simo Huang, Hong Lei, Zhan Yang, Yuxin Su, Xiaoming He, Qinghe Zhao, Yong Wang, Wei Liu, Liuyu Huang

**Affiliations:** ^1^Chinese People's Liberation Army Institute for Disease Control and Prevention, Beijing, China; ^2^Department of Clinical Laboratory, 309th Hospital of Chinese People's Liberation Army, Beijing, China

**Keywords:** colistin, the *mcr-1* gene, *Enterobacteriaceae*, LAMP, rapid detection

## Abstract

The emergence of the plasmid-encoded colistin-resistance gene *mcr-1* in *Enterobacteriaceae* represents a new threat to the treatment of infection in the clinical setting. A sensitive and rapid molecular method for detection of the *mcr-1* gene in clinical isolates is needed to control the spread of this gene. In this study, we established a loop-mediated isothermal amplification (LAMP) assay for rapid detection of the *mcr-1* gene. This assay was applied to cultured bacteria and spiked human stools. Real-time monitoring of turbidity and chromogenic visualization were used to assess the reaction results. The specificity and sensitivity of the primers in the LAMP reactions for detection of the *mcr-1* gene were determined. All 20 clinically resistant isolates without the *mcr-1* gene tested negative, indicating the high specificity of the LAMP primers. The sensitivity of LAMP, with a detection limit of 0.2 pg/μL DNA, was 10-fold greater than that of polymerase chain reaction (PCR). The assay was also conclusive when applied to human stools spiked with *mcr-1*-positive *Escherichia coli*. During clinical screening in a major hospital in Beijing, China, seven isolates were identified as positive from the 556 *Enterobacteriaceae* isolates. In conclusion, the LAMP assay we developed was useful for detection of the *mcr-1* gene in the clinical setting.

## Introduction

Colistin is a polymyxin antibiotic that is considered as the final line of treatment for multidrug-resistant gram-negative bacteria (Li et al., 2006). In 2015, the first plasmid-mediated colistin-resistance gene, *mcr-1*, was identified in *Enterobacteriaceae* isolates of human and animal origin (Liu et al., 2016). Subsequently, the *mcr-1* gene has been found in many countries around the world, as demonstrated by screening of early isolated strains and whole-genome sequencing of bacteria (Arcilla et al., 2016; Mulvey et al., 2016; Webb et al., 2016). Moreover, a report of a carbapenem-resistant *Enterobacteriaceae* (CRE) carrying the *mcr-1* gene highlighted the importance of this new global public health issue (Yao et al., 2016).

Recently, various molecular methods, including conventional polymerase chain reaction (PCR) and real-time PCR, have been established for detection of the *mcr-1* gene (Bontron et al., 2016; Liu et al., 2016). While these assays facilitate rapid, sensitive, and specific detection, they require specialized and expensive equipment. Therefore, another rapid and simple assay, termed loop-mediated isothermal amplification (LAMP), was developed to complement the existing PCR methods. LAMP is a nucleic acid detection technique that was first reported in 2000 and can be used to amplify DNA with Bst DNA polymerase under isothermal conditions, permitting autocycling strand displacement DNA synthesis (Notomi et al., 2000; Ushikubo, 2004). This method employs four to six primers recognizing a total of six to eight distinct sequences in the target DNA, giving the assay a high degree of specificity and amplification efficiency. LAMP is widely used for pathogen detection in infectious diseases (Parida et al., 2008).

Accordingly, in this study, we established and optimized a LAMP method for detection of the *mcr-1* gene. The specificity and sensitivity of the LAMP reaction for detection of the *mcr-1* gene were determined. In addition, the sensitivity of the LAMP assay for detection of the *mcr-1* gene in fecal samples was also tested.

## Materials and methods

### Bacterial strains and preparation of templates

In total, 20 multidrug-resistant bacteria without the *mcr-1* gene, as determined using the existing PCR method, were used as negative controls (Liu et al., 2016). Two isolates carrying the *mcr-1* gene, *Escherichia coli* 12452 and *E. coli* 12536, were screened by our laboratory in 2016 and used as positive controls. The information on these strains is shown in Table 1. Genomic DNA from bacteria was extracted using a one-step microbial DNA extraction method based on Chelex-100 (de Lamballerie et al., 1992). In addition, in order to assess the sensitivity of the LAMP assay, genomic DNA was also extracted from *E. coli* 12452 using a Wizard genomic DNA purification kit (Promega, Madison, WI, USA) according to the manufacturer's instructions. Purified DNA was then prepared by serial 10-fold dilutions to give concentrations ranging from 20 ng/μL to 0.02 pg/μL (Liu et al., 2012).

**Table 1:** Bacterial strains used in this study.

**No**.	**Species**	**Source**
1	*Escherichia coli* 12452	Clinical isolate
2	*Escherichia coli* 12536	Clinical isolate
3	*Escherichia coli* 12266	Clinical isolate
4	*Escherichia coli* 12278	Clinical isolate
5	*Klebsiella pneumonia* 12146	Clinical isolate
6	*Klebsiella pneumonia* 12163	Clinical isolate
7	*Acinetobacter baumannii* 12223	Clinical isolate
8	*Acinetobacter baumannii* 12250	Clinical isolate
9	*Stenotrophomonas maltophilia* 12285	Clinical isolate
10	*Stenotrophomonas maltophilia* 12455	Clinical isolate
11	*Pseudomonas aeruginosa* 12343	Clinical isolate
12	*Pseudomonas aeruginosa* 12352	Clinical isolate
13	*Enterobacter cloacae* 12105	Clinical isolate
14	*Enterobacter cloacae* 12211	Clinical isolate
15	*Proteus mirabilis* 12116	Clinical isolate
16	*Proteus mirabilis* 12407	Clinical isolate
17	*Serratia marcescens* 12208	Clinical isolate
18	*Serratia marcescens* 12330	Clinical isolate
19	*Salmonella typhimurium* 12306	Clinical isolate
20	*Salmonella typhimurium* 12161	Clinical isolate
21	*Shigella flexneri* 12221	Clinical isolate
22	*Shigella flexneri* 12169	Clinical isolate

### Primer design

The sequence of the *mcr-1* gene was downloaded from the GenBank database (accession number LC114018.1), and the gene-specific primers were then designed using the Primer Explorer (version 5) software (http://primerexplorer.jp/lampv5e/index.html). First, the forward inner primer (FIP), backward inner primer (BIP), outer forward primer (F3), and outer backward primer (B3) were designed. Then, additional loop primers (LF and LB) were designed to accelerate the amplification reaction (Notomi et al., 2000). All primers were synthesized by Sangon Biotech Co., Ltd. (Shanghai, China).

### Spiked stools and DNA extraction

Fifty milligrams of fresh feces from healthy individuals was suspended in 200 μL distilled water by vigorous shaking for 5 min. An amount of 10^8^ bacteria (corresponding to 0.2 OD_600_) or dilution was resuspended in 50 μL of 0.85% NaCl and spiked in 200 μL of human fecal suspension (Bontron et al., 2016). The total DNA was extracted from spiked stool samples with a QIAamp DNA Stool Mini Kit (Qiagen GmbH, Hilden, Germany) according to the manufacturer's instructions. DNA was recovered in 100 μL distilled water, and the LAMP assay was performed with 2 μL DNA template.

### LAMP reaction

The LAMP assay was performed in 25 μL reaction mixtures containing the following components: 20 mM Tris-HCl (pH 8.8), 10 mM KCl, 10 mM (NH4)_2_SO_4_, 8 mM MgSO_4_, 0.8 M betaine, 0.1% Tween-20, 1.4 mM of each dNTP, and 8 U Bst DNA polymerase. The amounts of primers needed for one reaction were 40 pmol for FIP and BIP, 20 pmol for LF and LB, and 5 pmol for F3 and B3. Finally, an appropriate amount of genomic DNA template was added to the reaction tube (Liu et al., 2012). The mixture was covered with 25 μL wax and incubated for 60 min in a dry bath incubator with a controlled temperature. Temperature was optimized by testing at 60–67°C.

LAMP assay amplification products were detected using two different methods: turbidimetry and visual detection. For monitoring of turbidity, real-time amplification by the LAMP assay was monitored using a Loopamp real-time turbidimeter (LA320-C; Eiken Chemical Co. Ltd., Tokyo, Japan). For visual detection, manganese ion (Mn^2+^) and calcein, a fluorescent metal indicator, were added to the reaction mixture, which appeared orange because calcein was quenched by Mn^2+^. For a positive reaction, Mn^2+^ formed a magnesium pyrophosphate precipitate (an insoluble salt) during amplification, and the color of the reaction system changed from orange to green. For a negative reaction, there was no color change because amplification did not occur (Mori et al., 2001).

### PCR assay

PCR was performed to compare assay sensitivities for detection of the *mcr-1* gene using the previously reported CLR5-F and CLR5-R primers (Liu et al., 2016). PCR tubes were set up using 12.5 μL PCR MasterMix reagents (Tiangen Biotech Co., Ltd., Beijing, China), 1 μM CLR5-F and CLR5-R primers (0.4 μM), and 1 μL DNA template in a final volume of 25 μL. The thermal cycler parameters were 3 min at 95°C; 30 cycles of 30 s at 95°C, 30 s at 55°C, and 30 s at 72°C; followed by 5 min at 72°C. The PCR-amplified products were analyzed by 1% agarose gel electrophoresis and stained with GelRed nucleic acid gel stain (Biotium, Hayward, CA, USA). Images were documented using a Bio-Rad Gel Doc EQ imaging system (Bio-Rad, Hercules, CA, USA).

## Results

### Ethics statement

All volunteers provided written, informed consent to participate in this study, which was reviewed and approved by the ethics committee of the Academy of Military Medical Sciences, China. All experiments were performed in accordance with relevant guidelines and regulations.

### Primer screening

**Figure 1:**
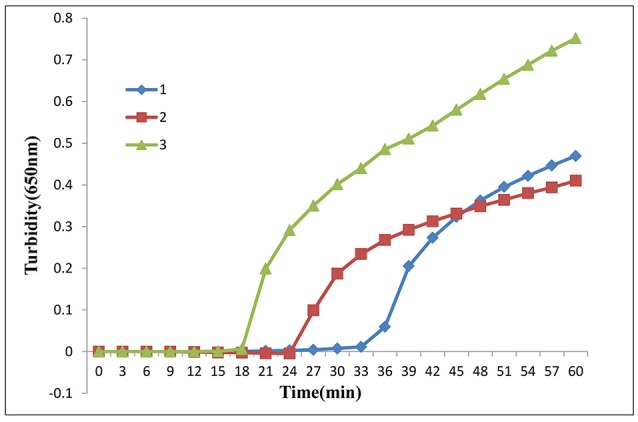
LAMP assay primer screening. Three sets of primers amplified the target gene under the same conditions. Turbidity was monitored by a Loopamp real-time turbidimeter at 650 nm every 6 s.

Five sets of primers for detection of the *mcr-1* gene were designed. According to the primer parameters and variants of *mcr-1* gene mutations (Thanh et al., 2016), we excluded the fourth and fifth sets of primers and evaluated the other three sets of primers. Under the same reaction conditions, three turbidity curves were observed, indicating that all three primers had the ability to amplify the target fragment. Through this analysis, we found that the third set of primers allowed for completion of the amplification in the shortest time (Figure 1). Primers in the third primer set (Table 2) were chosen as the final primers for *mcr-1* gene detection by LAMP. For more information on the five sets of primers, see the Supplementary Materials.

**Table 2:** Primers used for amplification of the *mcr-1* gene.

**Primer**	**Sequence (5′-3′)**	**Location**
3-F3	ACAAGCAACCAAGCCTGAT	681–699
3-B3	GCGCCCAGATAGCTGAAC	876–893
3-FIP	GAAGCTGACATGATCGGCGCGCGTAAGCCACGCCTAGTG	745–765, 703–720
3-BIP	TTCCCACAGCTTGCCAAGATCGGCACAGAATACGCCGTCG	787–808, 851–868
3-LB	AGCAATGTCACATCGTGCG	826–844

### Optimization of temperature for the lamp assay

**Figure 2:**
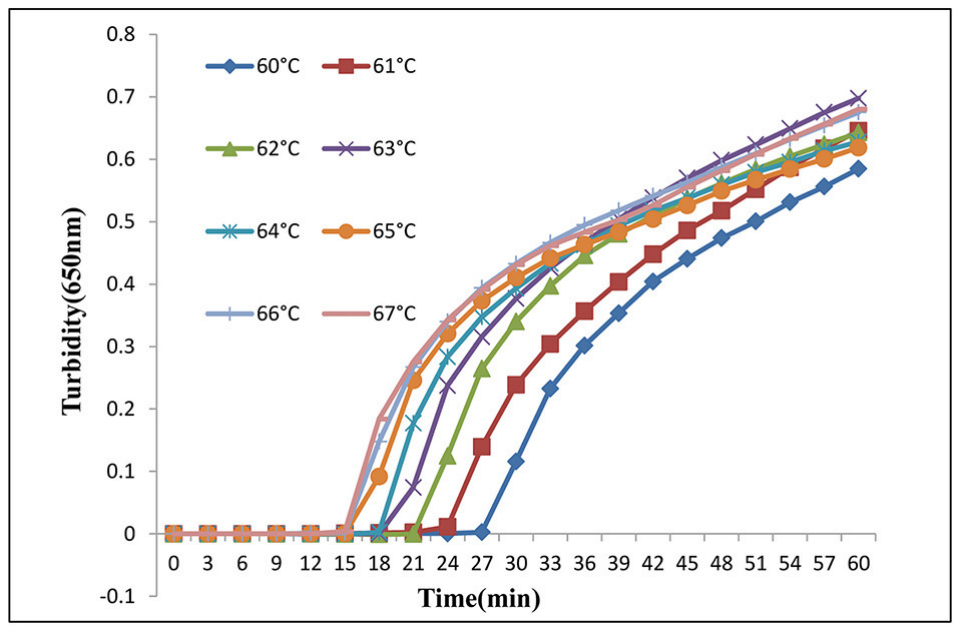
LAMP assay temperature optimization. Different temperatures (60–67°C at 1°C intervals) were tested and 66°C was chosen as the optimal temperature for LAMP amplification.

Various reaction temperatures ranging from 60 to 67°C were employed at 1°C intervals to optimize the amplification. As shown in Figure 2, LAMP assay amplification occurred in the range of 60–67°C, and shorter reaction times were observed at 65, 66, and 67°C compared with those at other temperatures. We chose 66°C as the optimum temperature due to the higher peak and stronger enzyme activity in the LAMP assay (the optimum temperature for enzymatic activity of the *Bst* DNA polymerase was 65°C). Finally, we chose 66°C as the reaction temperature for later experiments.

### Specificity of the lamp assay for the *mcr-1* gene

**Figure 3:**
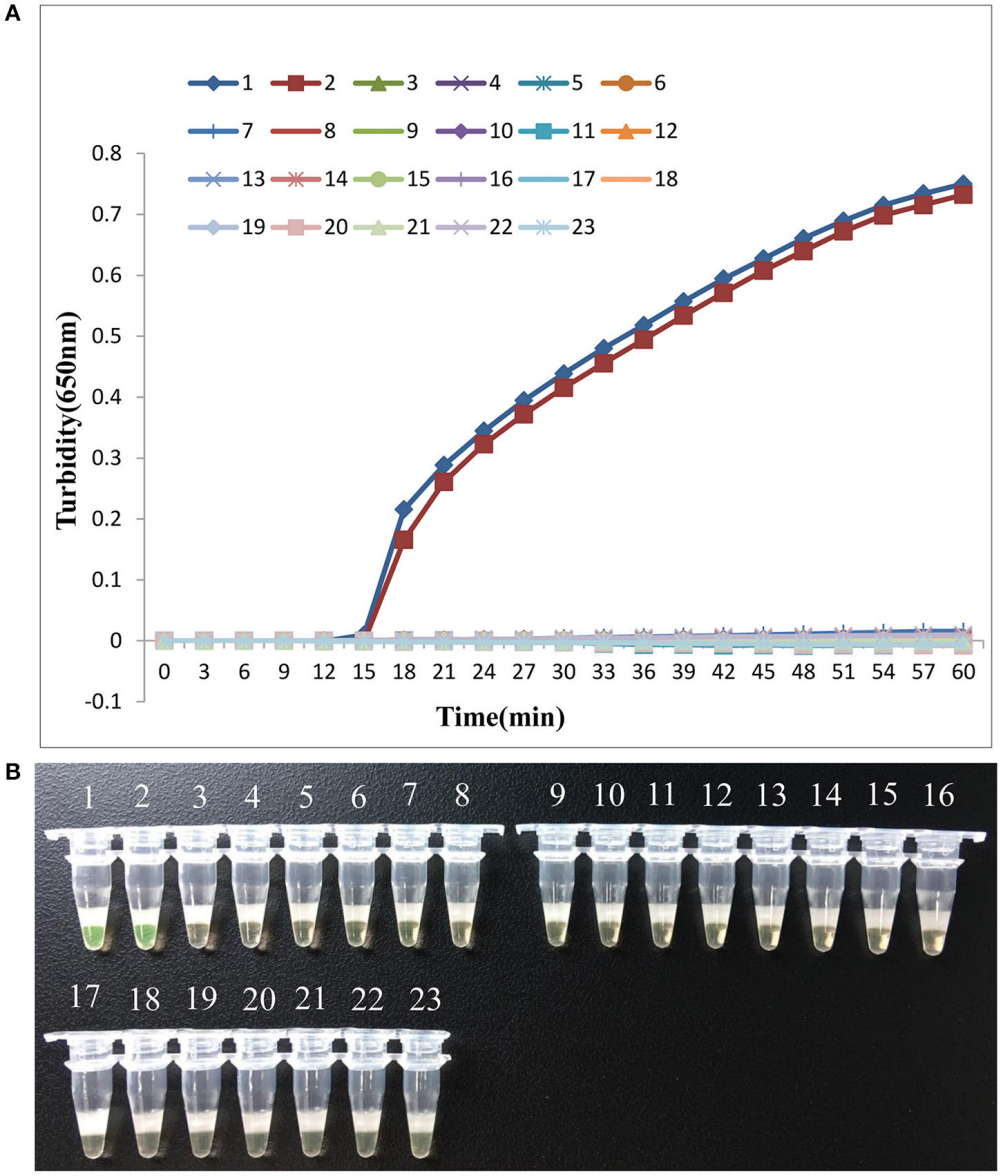
LAMP assay specificity. Specificity of the LAMP method for detecting the *mcr-1* gene by real-time turbidimeter **(A)** or visual detection based on a fluorescent metal indicator **(B)**. Amplification was performed at 66°C for 60 min. 1, *E. coli* 12452; 2, *E. coli* 12536; 3, *E. coli* 12266; 4, *E. coli* 12278; 5, *K. pneumonia* 12146; 6, *K. pneumonia* 12163; 7, *Acinetobacter baumannii* 12223; 8, *A. baumannii* 12250; 9, *Stenotrophomonas maltophilia* 12285; 10, *S. maltophilia* 12455; 11, *Pseudomonas aeruginosa* 12343; 12, *P. aeruginosa* 12352; 13, *Enterobacter cloacae* 12105; 14, *E. cloacae* 12211; 15, *Proteus mirabilis* 12116; 16, *P. mirabilis* 12407; 17, *Serratia marcescens* 12208; 18, *S. marcescens* 12330; 19, *Salmonella typhimurium* 12306; 20, *S. typhimurium* 12161; 21, *Shigella flexneri* 12221; 22, *Shigella flexneri* 12169; 23, negative control (double-distilled water).

Next, we analyzed the specificity of LAMP detection of the *mcr-1* gene. As shown in Figure 3, only when *E. coli* 12452 and *E. coli* 12536 were used as the templates, both turbidity and visual detection appeared positive. Importantly, 20 multidrug-resistant bacteria without the *mcr-1* gene, including the blank control, tested negative, indicating that the LAMP assay was highly specific for the *mcr-1* gene.

### Sensitivity of the lamp assay for the *mcr-1* gene

**Figure 4:**
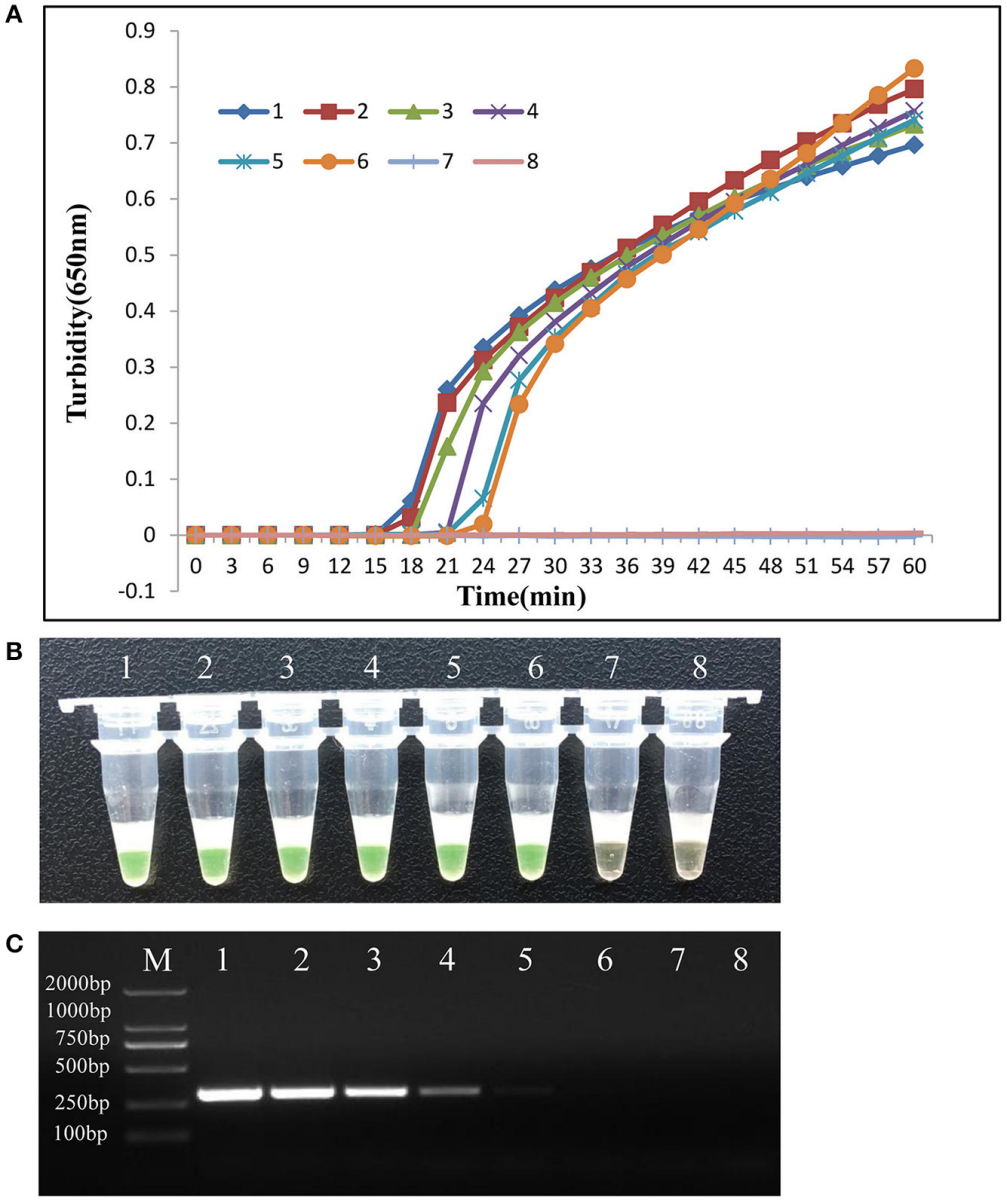
LAMP assay sensitivity. Comparative sensitivities of the LAMP assay **(A)** and **(B)** and traditional PCR **(C)** for detection of the *mcr-1* gene. 1–7:10-fold serial dilution of genomic DNA extracted from *E. coli* 12452; 8: negative control (double-distilled water). The expected PCR product size was 309 bp.

To determine the sensitivity of primers in LAMP detection of the *mcr-1* gene, purified genomic DNA was extracted from *E. coli* 12452 and subjected to serial 10-fold dilutions. PCR was also carried out with the same amount of purified DNA template. As shown in Figure 4, the six reaction curves of real-time turbidity indicated that six serial 10-fold dilutions of the template were detected (20 ng/μL to 0.2 pg/μL), consistent with results of the visual method (positive reactions turned green, whereas negative reactions remained orange). The detection limit of the LAMP assay for the *mcr-1* gene was 0.2 pg/μL, which was 10 times more sensitive than conventional PCR.

### Evaluation of the lamp assay in spiked stool samples

Because the *mcr-1* gene is often detected in human and cattle feces, LAMP assays were applied to human stools spiked with different amounts of *mcr-1*-positive bacteria. PCR was also carried out as a control. The results showed that the detection limit of the LAMP assay for human stools was 10^3^ bacteria, which was also 10 times more sensitive than conventional PCR. Thus, LAMP assays can be efficiently applied to detection of the *mcr-1* gene in fecal samples.

### Detection of the *mcr-1* gene in clinical isolates

A LAMP-based surveillance method for detection of the *mcr-1* gene in clinical isolates was conducted in a local hospital in Beijing, China. In total, 556 multidrug-resistant *Enterobacteriaceae* were collected in 2016 from patients with suspected infections; these bacteria had been screened for the *mcr-1* gene by PCR using the above primers (Liu et al., 2016).

Subsequently, the results of LAMP assays showed that seven *mcr-1*-positive isolates, including six *E. coli* and one *Klebsiella pneumoniae*, were detected from 556 bacteria. These results were consistent with the results of the PCR assay. Finally, these *mcr-1*-positive isolates were validated again by PCR-based sequencing. Thus, these findings demonstrated that LAMP assays were well suited for large-scale clinical screening.

## Discussion

In Europe and the United States of America, clinical infections caused by multidrug-resistant or pan-resistant *Acinetobacter baumannii* and *Pseudomonas aeruginosa* are often treated with topical polymyxins. These drugs are also used to treat severe infections caused by CRE combined with other types of antibiotics (Falagas et al., 2010). However, the use of antibiotics in agriculture and the nonstandard use of antibiotics in the clinic have accelerated the emergence of drug-resistant bacteria. In the second half of 2015, the first plasmid-mediated colistin-resistance gene, *mcr-1*, was reported in China, attracting the attention of scientists and physicians worldwide (Liu et al., 2016). By the second half of 2016, *mcr-1*-positive isolates were found in more than 30 countries around the world (Al-Tawfiq et al., 2017). The *mcr-1* gene was found in bacteria isolated from various food animals, the environment (river water), various types of meats and vegetables, and infected patients and asymptomatic human carriers, including international travelers (Bernasconi et al., 2016; Hu et al., 2016; Liu et al., 2016; Zurfuh et al., 2016). The *mcr-1* gene has been found in *Enterobacteriaceae*, mostly *E. coli* and *K. pneumonia* (Quan et al., 2017). The positive rate of animal *mcr-1*-positive isolates is much higher than that of clinical isolates (Liu et al., 2016). Therefore, *mcr-1*-positive strains can spread in hospitals, causing outbreaks and epidemics of nosocomial infections. Thus, CRE carrying the *mcr-1* gene will undoubtedly become a major global public health issue (Falgenhauer et al., 2016; Yao et al., 2016).

Detection of drug resistance genes is critical for the prevention of bacterial resistance. Conventional and real-time PCR methods have been established for detection of the *mcr-1* gene (Bontron et al., 2016; Liu et al., 2016). However, these methods require expensive equipment. Thus, to complement existing methods, we established a simple and efficient LAMP method. The LAMP assay was found to be highly specific for the *mcr-1* gene, and the detection limit of the LAMP assay for the *mcr-1* gene was 0.2 pg/μL, which was 10 times more sensitive than that of conventional PCR. In addition, the LAMP assay could be efficiently applied to detection of the *mcr-1* gene in fecal samples.

## Conclusion

In this study, we established a LAMP detection method for the *mcr-1* gene; this method was found to be a rapid, specific, sensitive, and cost-effective tool for the identification of resistance genes. We anticipate that this method may be used routinely in the screening of clinically resistant bacteria. To the best of our knowledge, this is the first detailed report on the application of LAMP for detection of the *mcr-1* gene.

## Author contributions

LH and WL designed experiments; DZ, SH, and HL carried out experiments; ZY, YS, and XH analyzed experimental results; QZ and YW collected clinical isolates and related information; DZ wrote the manuscript.

### Conflict of interest statement

The authors declare that the research was conducted in the absence of any commercial or financial relationships that could be construed as a potential conflict of interest.
