# Supplementary material for: Sensitive and Rapid Detection of the Plasmid-Encoded Colistin-Resistance Gene mcr-1 in Enterobacteriaceae Isolates by Loop-Mediated Isothermal Amplification
Source: Front Microbiol. 2017 Nov 29;8:2356. doi: 10.3389/fmicb.2017.02356 (PMC5712548; doi:10.3389/fmicb.2017.02356)
Supplement: Supplementary file 1 [file Image1.PDF]

PrimerExplorer V5 Software

1. Push "Primer Information" button to download Primer Information format file for loop primer designing.
2. Push "Save" button to download the primer information in the screen display layout.

DesignId 170428100017

Primer Information | Save

1 ID:33 dimer(minimum)dG=-2.18

label	5'pos	3'pos	len	Tm	5'dG	3'dG	GCrate	Sequence
F3	1382	1401	20	59.67	-3.79	-5.90	0.50	TGTATGTCAGCGATCATGGC
B3	1574	1591	18	59.78	-7.87	-4.33	0.56	CCGCGGTGACATCAAACA
FIP			42					CTGCGCTGTTCTTTTGGTGCAA-GAAAGTCTGGGTGAGAACGG
BIP			40					GTGCCTGCATTTTTCTGGACGG-TGATCGCGTCATGGGTCA
F2	1402	1421	20	59.73	-4.01	-5.79	0.55	GAAAGTCTGGGTGAGAACGG
F1c	1451	1472	22	64.51	-7.42	-5.57	0.50	CTGCGCTGTTCTTTTGGTGCAA
B2	1538	1555	18	60.36	-5.09	-5.86	0.56	TGATCGCGTCATGGGTCA
B1c	1474	1495	22	64.40	-6.24	-6.63	0.55	GTGCCTGCATTTTTCTGGACGG

Primer Information | Save

2 ID:24 dimer(minimum)dG=-2.34

label	5'pos	3'pos	len	Tm	5'dG	3'dG	GCrate	Sequence
F3	1020	1039	20	59.72	-4.51	-5.18	0.40	AAAAGCGCAATTTGCCGATT
B3	1217	1236	20	60.94	-7.08	-4.16	0.45	TGGCGTGAATTTGGCAAACT
FIP			40					CGAGCATACCGACATCGCGG-TAAATCCGCGACCAACAACG
BIP			39					TTGTCGCTGCCAATAACGGCA-ATACGCAGGCCCGTGATT
F2	1041	1060	20	60.59	-2.75	-5.49	0.50	TAAATCCGCGACCAACAACG
F1c	1092	1111	20	64.92	-6.43	-7.71	0.65	CGAGCATACCGACATCGCGG
B2	1180	1197	18	60.84	-5.30	-4.06	0.56	ATACGCAGGCCCGTGATT
B1c	1127	1147	21	65.17	-5.35	-7.08	0.52	TTGTCGCTGCCAATAACGGCA

Primer Information | Save

3 ID:13 dimer(minimum)dG=-2.38

label	5'pos	3'pos	len	Tm	5'dG	3'dG	GCrate	Sequence
F3	681	699	19	59.39	-5.40	-4.74	0.47	ACAAGCAACCAAGCCTGAT
B3	876	893	18	59.69	-8.37	-4.51	0.61	GCGCCCAGATAGCTGAAC
FIP			39					GAAGCTGACATGATCGGCGCG-CGTAAGCCACGCCTAGTG
BIP			40					TTCCCACAGCTTGCCAAGATCG-GCACAGAATACGCCGTCG
F2	703	720	18	59.22	-4.51	-4.07	0.61	CGTAAGCCACGCCTAGTG
F1c	745	765	21	65.87	-5.09	-8.70	0.62	GAAGCTGACATGATCGGCGCG
B2	851	868	18	60.14	-5.90	-6.96	0.61	GCACAGAATACGCCGTCG
B1c	787	808	22	65.15	-5.37	-4.92	0.55	TTCCCACAGCTTGCCAAGATCG

Primer Information | Save

4 ID:1 dimer(minimum)dG=-2.33

label	5'pos	3'pos	len	Tm	5'dG	3'dG	GCrate	Sequence
F3	5	24	20	59.80	-6.65	-5.66	0.50	TGCAGCATACTTCTGTGTGG
B3	222	241	20	59.38	-4.58	-5.80	0.45	TCAACACAGGCTTTAGCACA
FIP			39					GTAAGATTGGCGGTCGCGGTC-GCTCGGTCAGTCCGTTTG
BIP			42					ACCTATCCCATCGCGGACAATC-CGTGGTGATCAGTAGCATCG
F2	32	49	18	59.75	-6.87	-5.10	0.61	GCTCGGTCAGTCCGTTTG
F1c	78	98	21	65.35	-3.59	-7.03	0.62	GTAAGATTGGCGGTCGCGGTC
B2	182	201	20	59.85	-6.33	-6.08	0.55	CGTGGTGATCAGTAGCATCG
B1c	121	142	22	64.01	-3.96	-4.06	0.55	ACCTATCCCATCGCGGACAATC

Primer Information | Save

5 ID:6 dimer(minimum)dG=-2.43

label	5'pos	3'pos	len	Tm	5'dG	3'dG	GCrate	Sequence
F3	251	268	18	60.81	-3.15	-6.19	0.56	TAATCATGGGCGCGGTGA
B3	467	484	18	60.37	-5.09	-6.42	0.61	CGATCAAGCCCAATCGGC
FIP			41					GTCTCGGCTTGGTCGGTCTGTA-CTGACACTTATGGCACGGT
BIP			42					TGGTTTGGGTGTGCTACCAAGT-CCCCAAGTCGGATAATCCAC
F2	281	299	19	59.05	-4.91	-6.33	0.53	CTGACACTTATGGCACGGT
F1c	329	350	22	65.66	-5.53	-3.99	0.59	GTCTCGGCTTGGTCGGTCTGTA
B2	433	452	20	59.42	-5.96	-4.90	0.55	CCCCAAGTCGGATAATCCAC
B1c	390	411	22	64.35	-4.67	-5.00	0.50	TGGTTTGGGTGTGCTACCAAGT
